# Salivary bacterial signatures in depression-obesity comorbidity are associated with neurotransmitters and neuroactive dipeptides

**DOI:** 10.1186/s12866-022-02483-4

**Published:** 2022-03-14

**Authors:** Gajender Aleti, Jordan N. Kohn, Emily A. Troyer, Kelly Weldon, Shi Huang, Anupriya Tripathi, Pieter C. Dorrestein, Austin D. Swafford, Rob Knight, Suzi Hong

**Affiliations:** 1 Department of Psychiatry, University of California San Diego, La Jolla, CA 92093 USA; 2 Center for Microbiome Innovation, University of California San Diego, La Jolla, CA 92093 USA; 3 Skaggs School of Pharmacy and Pharmaceutical Sciences, University of California San Diego, La Jolla, CA 92093 USA; 4 Department of Pediatrics, University of California San Diego, La Jolla, CA 92093 USA; 5 Collaborative Mass Spectrometry Innovation Center, University of California San Diego, La Jolla, CA 92093 USA; 6 Department of Computer Science and Engineering, University of California San Diego, La Jolla, CA 92093 USA; 7 Department of Bioengineering, University of California San Diego, La Jolla, CA 92093 USA; 8 Herbert Wertheim School of Public Health and Human Longevity Science, University of California San Diego, La Jolla, CA 92093 USA

**Keywords:** Oral microbiome, Depression, Obesity, Host inflammation, Host-microbe interactions, Neuroactive molecules

## Abstract

**Background:**

Depression and obesity are highly prevalent, often co-occurring conditions marked by inflammation. Microbiome perturbations are implicated in obesity-inflammation-depression interrelationships, but how the microbiome mechanistically contributes to pathology remains unclear. Metabolomic investigations into microbial neuroactive metabolites may offer mechanistic insights into host-microbe interactions. Using 16S sequencing and untargeted mass spectrometry of saliva, and blood monocyte inflammation regulation assays, we identified key microbes, metabolites and host inflammation in association with depressive symptomatology, obesity, and depressive symptomatology-obesity comorbidity.

**Results:**

Gram-negative bacteria with inflammation potential were enriched relative to Gram-positive bacteria in comorbid obesity-depression, supporting the inflammation-oral microbiome link in obesity-depression interrelationships. Oral microbiome was more highly predictive of depressive symptomatology-obesity co-occurrences than of obesity or depressive symptomatology independently, suggesting specific microbial signatures associated with obesity-depression co-occurrences. Mass spectrometry analysis revealed significant changes in levels of signaling molecules of microbiota, microbial or dietary derived signaling peptides and aromatic amino acids among depressive symptomatology, obesity and comorbid obesity-depression. Furthermore, integration of the microbiome and metabolomics data revealed that key oral microbes, many previously shown to have neuroactive potential, co-occurred with potential neuropeptides and biosynthetic precursors of the neurotransmitters dopamine, epinephrine and serotonin.

**Conclusions:**

Together, our findings offer novel insights into oral microbial-brain connection and potential neuroactive metabolites involved.

**Supplementary Information:**

The online version contains supplementary material available at 10.1186/s12866-022-02483-4.

## Background

Depression and obesity are common, debilitating, and frequently co-occurring chronic conditions with increasing incidences globally [1]⁠. Nearly 39% of the adult population are overweight and 13% are obese worldwide (WHO, 2016), while 5% of the world population are affected by mood disorders (WHO, 2017) [2, 3]⁠. The relationship between obesity and depression is often bidirectional [4]⁠, as prevalence of depression among individuals with obesity is significantly higher than that in the general population [5, 6]. Conversely, individuals with depression are more likely to develop obesity compared to non-depressed individuals [7]⁠. Despite the advent of antidepressant drugs and their long-term usage in clinical treatment, the majority of patients with depression are treatment-refractory, and obesity may further reduce the efficacy of antidepressants [8]⁠. Furthermore, comorbid depression and obesity are strongly associated with several diseases such as type 2 diabetes mellitus, cardiovascular diseases, chronic kidney disease and cancer, reducing both longevity and quality of life [2, 9]⁠. Therefore, obesity and depression, and their co-occurrence, pose a major public health concern worldwide.

Inflammatory dysregulation is a common pathogenic mechanism underlying the co-occurrence of depression and obesity, as both are associated with chronic low-grade inflammation [10, 11]⁠. Individuals with obesity and depression evidence increased concentrations of peripheral and central inflammatory cytokines and acute phase reactants, such as interleukin (IL)-6, tumor necrosis factor alpha (TNF-α), and C-reactive protein (CRP) [11, 12]⁠⁠. In obesity, macrophages accumulate in adipose tissue leading to local and systemic inflammation [13, 14]⁠, which can contribute to depressive symptoms via multiple mechanisms, such as by decreasing neurotransmitter availability, and by potentiating neuroinflammatory processes such as microglial activation and peripheral monocyte trafficking to the central nervous system (CNS) [10, 15, 16]⁠. It should be noted, however, that inflammation has been shown to underlie only a subset of depression cases [17]⁠, hence the conceptualization of a theoretical immuno-metabolic *subtype* of major depressive disorder [18]. Nonetheless, inflammatory dysregulation remains a central mechanism underlying the co-occurrence of depression and obesity, and this is likely relevant to sub-clinical depressive symptomatology. To this end, our previous work has demonstrated that even in individuals without clinical diagnosis of depression, higher depressive symptom scores, obesity, and downregulated glucocorticoid and adrenergic receptor-mediated cellular inflammatory control are interrelated [19–21].

Although psychological stress, host genetics and environmental factors have been shown to contribute to obesity and depression, recently, the human microbiome (i.e., collection of diverse microorganisms and their genetic material) and metabolome (i.e., a large collection of structurally diverse metabolites) have been implicated in processes of energy homeostasis, mood and behavior, and immune regulation, and may therefore offer a novel mechanism underlying the co-occurrence of depression and obesity [2]. Animal studies of obesity have shown that depletion of members of *Bifidobacterium*, *Lactobacillus*, and *Akkermansia* are associated with weight gain, increased inflammation, increased depressive behavior and changes in neural circuitry [22, 23]⁠. Animal studies have also shown that increased permeability in the intestinal barrier and the blood-brain barrier (BBB) are associated with increased plasma lipopolysaccharide (LPS) levels [22–24]⁠ and neuroinflammation [23]⁠. Altogether, these studies suggest that increased intestinal barrier permeability and subsequent translocation of gut bacterial endotoxin, particularly LPS from Gram-negative bacterial cell walls into systemic circulation, may cause metabolic endotoxemia, leading to immune cell activation and production of pro-inflammatory cytokines such as IL-1β, IL-6 and TNF-α [2]. Increased dietary saturated fatty acids are also known to stimulate adipocytes and macrophages for pro-inflammatory cytokine production, and adipose tissue accumulates adipose tissue-resident macrophages, which can further promote inflammation [25]⁠. These processes may further contribute to increased BBB permeability, leading to accumulation of pro-inflammatory cytokines and immune cells in the brain, which can potentiate neuroinflammatory processes, and therefore serve as a potential mechanism underlying the occurrence of depressive symptoms in the context of obesity.

It is to be noted that human microbiome studies in depression and obesity, and indeed in health and disease, have focused largely on the ecosystem of the distal gut, while few studies have examined the microbial ecology of the oral cavity outside of oral-related conditions such as dental caries (i.e., tooth decay) and periodontitis (i.e., severe gum inflammation). The oral cavity, an entry portal to both the digestive and respiratory tracts, contains the most diverse microbial community after the gut, harboring more than 700 unique bacterial species with at least 150 specialized bacterial species per mouth [25, 26]. More than 60% of the microbial species found in the oral cavity have been shown to be potentially transmitted to the gut, suggesting that oral cavity is a reservoir for gut microbial strains in shaping the gut microbiome in health and disease [27]. Dysregulation of the unique microbe-microbe and microbe-host interactions in the oral ecosystem has been associated with systemic inflammatory diseases such as inflammatory bowel syndrome [28, 29]⁠ beyond an array of oral diseases. In addition, oral microbiota have also been associated with several neurological diseases, such as Alzheimer’s disease (AD) [30]⁠, multiple sclerosis [31]⁠ and Parkinson’s disease [32]⁠. Previously, our group found that salivary microbial diversity and diurnal variability were associated with both peripheral proinflammatory cytokine levels and psychological distress in this cohort on which this study is based [33]. The intimate link between the oral microbiota and systemic human diseases, as evidenced by aforementioned studies suggests that the oral cavity is likely a promising site for gaining insight into the pathophysiology of depression-obesity comorbidity. Moreover, the oral cavity is easily accessible via non-invasive as well as ‘on-demand’ collection of saliva samples for multi-omics applications.

While mechanisms linking the oral microbiota to the brain (i.e. “oral-brain axis”) remain largely unknown [34, 35]⁠, recent studies have speculated several transmission routes of how oral bacteria may reach the brain and influence neuro-immune activity and inflammation [36]⁠. For instance, routine dental procedures such as flossing, brushing and cleaning may cause oral bacteria to enter the blood circulation and cause bacteremia [37]⁠, and some of these microbes may traverse the BBB. Alteration in the permeability of the BBB may also expose the brain to bacterial metabolites triggering an inflammatory response, which in turn alters functioning of the CNS. For example, *Porphyromonas gingivalis*, a resident oral bacterium and a keystone pathogen in periodontitis has been found in the brain of AD patients [30]⁠ as well as neurotoxic proteases i.e., gingipains produced by *P. gingivalis* [30]⁠.

A recent study has shown that human gut bacteria encode at least 56 gut-brain metabolic pathways, which encompass both known and novel microbial pathways for synthesis and degradation of a number of neurotransmitters that have potential to cross the intestinal barrier and BBB [35]⁠. A subset of these gut-brain pathway effectors, for instance dopamine, glutamate, tryptophan and gamma-aminobutyric acid (GABA) were either enriched or depleted in patients with major depression [35]⁠. In particular, tryptophan metabolic pathways have been shown to be widely distributed across human gut bacterial species [35]⁠. Intriguingly, the majority of these gut bacterial species with neuroactive potential are also found to be residents of the oral cavity [25]⁠. However, to what extent these bacterial species can truly biosynthesize neurotransmitters within the host, either in the gut or the oral cavity, remains unknown. Thus, utilization of metabolomics offers a functional readout of both host and microbial phenotypes encoded in the genome [38, 39]⁠, and in conjunction with microbiome analyses, can provide mechanistic insights, yet current knowledge is greatly limited. In particular, microbial specialized metabolites have been shown to be canonical mediators of microbe-microbe and microbe-host interactions, and the most predominant specialized metabolites are of great interest for understanding the mechanisms of these interactions at the molecular level [38–40]⁠⁠. In this regard, the vast and highly diverse array of short peptides shown to play key roles in bacterial cell signaling [41], immune modulation, and neuroactive metabolism [42–44]⁠ remains largely unexplored. A recent study has shown that depletion of a variety of structurally uncharacterized dipeptides are associated with inflammatory bowel disease, a chronic inflammatory condition of the gastrointestinal tract [45]⁠. These observations prompted us to hypothesize that neurotransmitters and dipeptides likely have pivotal roles in obesity-inflammation-depression interrelationships.

In this study we aimed to investigate whether oral microbiota and small-molecule mediators of key microbe-microbe and microbe-host interactions differ by depressive symptomatology and obesity as well as their co-occurrence, and are influenced by inflammatory processes. We performed 16S rRNA gene-based sequencing of the oral microbiome and untargeted mass spectrometry of small-molecules from saliva, as well as host inflammation regulation profiles in blood from 60 participants.

## Results

### Participant characteristics

A total of 261 saliva samples collected from five time points across the day from 60 participants were analyzed (20 – 65 years): 50 participants had five; 51 had four, and 54 had three samples which were adjusted in analyses (See Statistical Analyses). Participants were categorized into the following four groups: non-obese (BMI < 30 kg/m2) and lower-depressive controls (*N* = 10 participants; *n* = 43 saliva samples; “controls”), obese (BMI ≥30 kg/m2) and lower-depressive (*N* = 18; *n* = 74; “Ob/lower-Dep”), non-obese and higher-depressive symptoms (*N* = 5; *n* = 22; “Non-ob/higher-Dep”), and obese and higher-depressive symptoms (*N* = 27; *n* = 122; “Ob/higher-Dep”). Sociodemographic characteristics are presented across participant groups (Table 1).

**Table 1:** Demographic and clinical characteristics of participants

Variable	Non-obese low depressive^a^	Obese low depressive^b^	Non-obese high depressive^c^	Obese high depressive^d^
Age	39 ± 12.2	38.9 ± 17.2	42.7 ± 10.5	43.5 ± 10.9
Sex (%female)	44	50	61.1	73.3
Race(%C/AA/Asn/NS)	72/16/12/0	37.5/37.5/12.5/12.5	55.6/16.7/27.8/0	46.7/40/13.3/0
BARIC	32.1 ± 10.2^d^	21.9 ± 6.2^c^	31.8 ± 9^cd^	25.3 ± 7.5^ac^
BMI (kg/m2)	25.1 ± 2.9^bd^	35.5 ± 4.7^ac^	26.6 ± 2.9^bd^	36 ± 4.7^ac^
BDI-T	0.5 ± 0.8^cd^	0.6 ± 0.7^cd^	7.9 ± 5.4^ab^	7.9 ± 5^ab^

Values presented as mean ± SD. Significant differences between groups were evaluated by Mann-Whitney test and presented as superscripts

*Abbreviations*: *C* Caucasian, *AA* African-American, *Asn* Asian, *NS* Mixed or not specified, *BARIC* Monocyte beta-adrenergic receptor-mediated inflammation control, *BMI* Body mass index, *BDI-T* Beck Depression Inventory (BDI-Ia) total score

### Obesity is associated with depressive symptomatology and inflammation

Given that individuals with a clinical diagnosis of depression and/or use of antidepressants were excluded from the study to focus on inflammation-related subclinical depressive symptoms in relation to obesity among otherwise healthy adults, BDI total scores (BDI-T) on average were low (median = 3; sd = 5; range = 0-22). The median value of BDI-T of ≥3 was used to divide participants with relatively ‘higher’ or ‘lower’ depressive symptoms in this non-clinical sample.

In all individuals, BMI was positively correlated with BDI-T scores (*r* = 0.29, *p* = 0.04), as well as cognitive-affective (*r* = 0.27, *p* = 0.03) and somatic symptom scores with small to medium effects (*r* = 0.22, *p* = 0.08) (Fig. S1). BARIC values, an indicator of neuro-inflammation regulation, were negatively correlated with BMI (*r* = − 0.38, *p* = 0.009), and an estimation of adipose tissue volume indicated by %trunk fat (*r* = − 0.25, *p* = 0.034) across all participants (Fig. S1). Age did not moderate any of these relationships, which is in agreement with previous findings [20]⁠. Altogether, obesity was significantly associated with both inflammation regulation and depressive symptoms. However, no significant associations were observed between BARIC and BDI scores in this study (Fig. S1).

### Oral microbiota differ based on obesity-depressive symptom groups and inflammation status

**Fig. 1:**
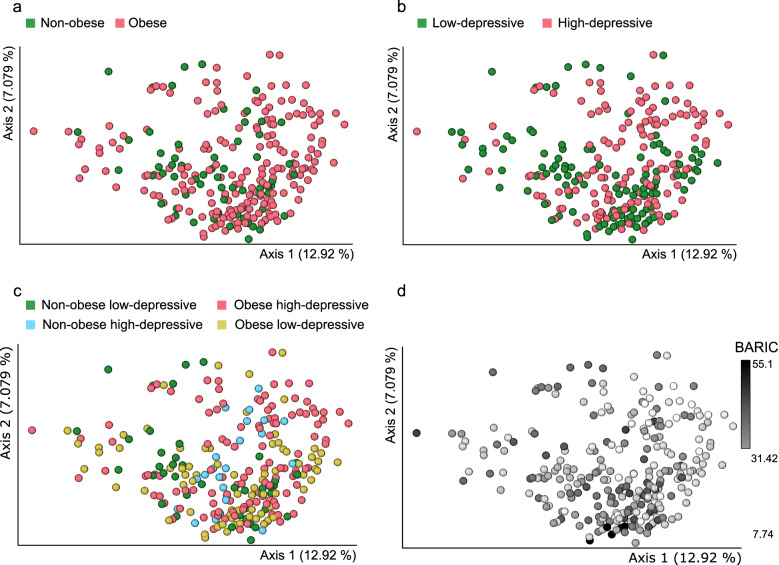
Principal coordinates analyses (PCoA) of oral bacterial communities in **a** non-obese and obese **b** low depressive and higher depressive **c** non-obese low-depressive, non-obese high-depressive, obese, and co-occurring obesity and depressive symptom groups, and **d** in inflammation status. Unweighted-UniFrac distances among samples were visualized using EMPeror. Significance of separation between the groups and further post-hoc pairwise comparisons between groups was tested by applying PERMANOVA test on the principal coordinates

Principal coordinates analysis (PCoA) and post-hoc pairwise comparisons of unweighted-UniFrac distances of samples revealed that oral microbiota composition was distinct by obesity (PERMANOVA pseudo-F = 1.9645, *p* = 0.003, Fig. 1a, Table 2), BDI-T (PERMANOVA pseudo-F = 2.4703, *p* = 0.001, Fig. 1b, Table 2) and across the four obesity-depressive symptom comorbid groups (i.e, Ctrl, Ob/lower-Dep, Non-ob/higher-Dep, Ob/higher-Dep) (Fig. 1c, Tables 2 and 3). Beta-diversity was also significantly differentiated based on the host inflammation across all participants (PERMANOVA pseudo-F = 4.7562, *p* < 0.001, Fig. 1d and Table 2). Significant beta-diversity differences were also observed by age, sex, and race but not by sampling time of day (Table 2). Phylogenetic alpha-diversity increased with inflammation (Faith’s PD: t = − 2.312, *p* = 0.025). Inflammation had slightly larger effects (R^2^ = 0.0181) on microbiome composition than obesity (R^2^ = 0.00756) and depressive symptomatology (R^2^ = 0.00948) (Table 2).

**Table 2:** Beta-diversity analysis of 16S derived ASVs across groups

Variable	*R*^2^	*F*	*P*-value
Age	0.01483	3.8849	0.001 ***
Sex	0.01042	2.7157	0.001 ***
Race	0.03504	3.0985	0.001 ***
Time of day	0.01125	0.7253	0.998
BARIC	0.0181	4.7562	0.001 ***
Obesity	0.00756	1.9645	0.003 **
Depressive symptomatology	0.00948	2.4703	0.001 ***
Obesity-depressive co-occurrences	0.02421	2.1175	0.001 ***

Asterisks indicate statistical significance of PERMANOVA test, *p* < 0.05. All *p*-values were generated based on 999 permutations

**Table 3:** Post-hoc pairwise comparisons of beta-diversity between groups

Pairwise contrasts	*R*^2^	*F*	*P*-value	FDR
Obese high-depressive x Non-obese low-depressive	0.01449	2.3819	0.001 ***	0.001 ***
Obese low-depressive x Non-obese low-depressive	0.01734	2.0294	0.001 ***	0.003 **
Non-obese high-depressive x Non-obese low-depressive	0.03364	2.1928	0.003 **	0.003 **
Obese low-depressive x Non-obese high-depressive	0.02118	2.034	0.004 **	0.004 **
Obese low-depressive x Obese high-depressive	0.01108	2.1632	0.003 **	0.002 **
Obese high-depressive x Non-obese high-depressive	0.01286	1.8369	0.002 **	0.002 **

Asteriks indicate statistical significance of PERMANOVA test, *p* < 0.05. All *p*-values were generated based on 999 permutations and then adjusted using the Benjamini–Hochberg method displayed in the table as FDR

### Oral microbiota is predictive of the host obesity-depressive symptomatology

**Fig. 2:**
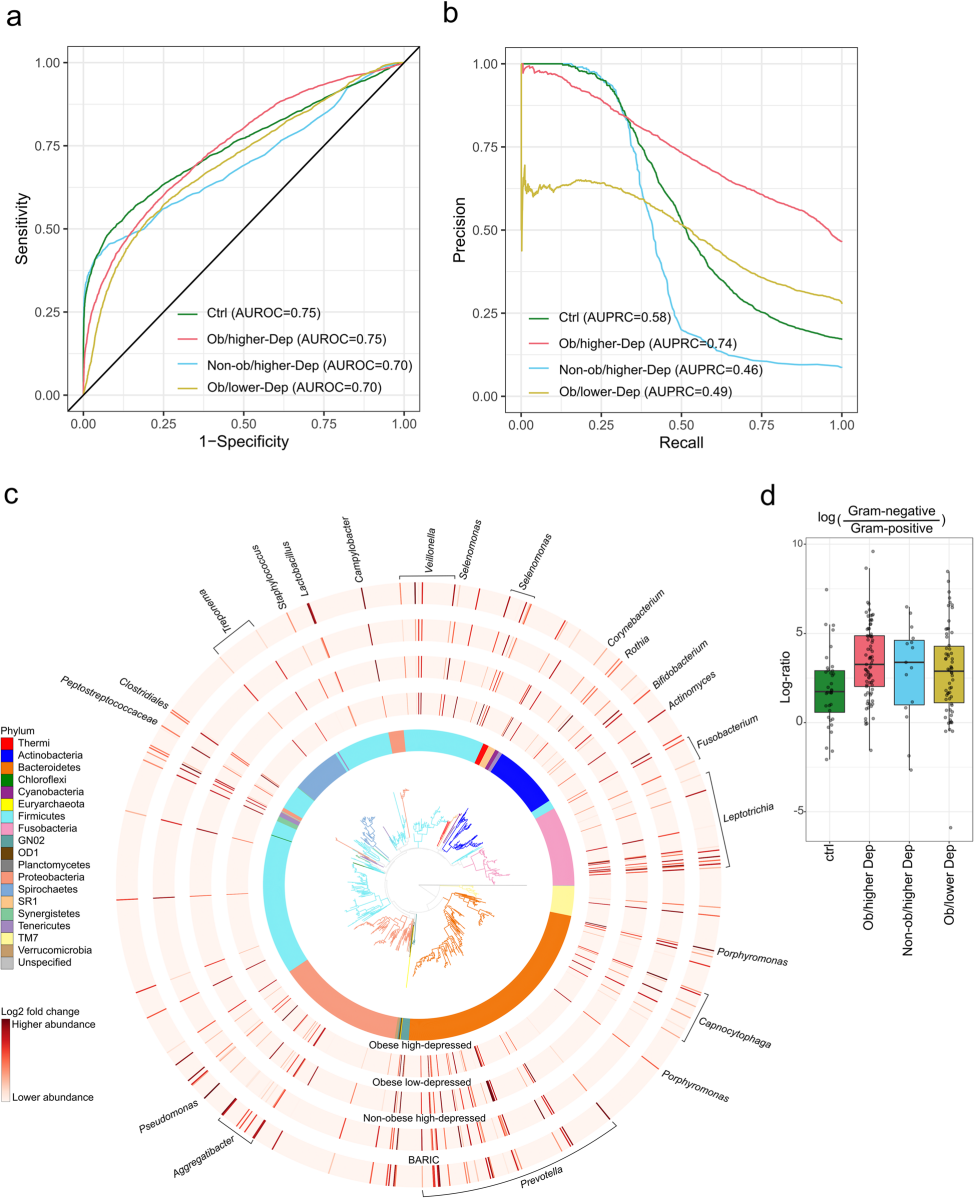
Oral microbiota is distinctly impacted by the host status in co-occurring obesity-depressive status. **a** Receiver operating characteristic curves (AUROC) illustrating classification accuracy of the random forest model across all groups (i.e. controls, Ob/lower Dep, Non-ob/higher-Dep, Ob/higher-Dep). **b** Area under precision recall curves (AUPRC) illustrating performance of the random forest model across all groups. **c** Phylogenetic distribution of the most differentially ranked taxa across the groups. Branches of the de novo phylogenetic tree and the innermost ring are colored by phyla. Each barplot layer represents log-fold change abundances of taxa within the group in comparison to the healthy controls i.e. Non-ob/lower-Dep. A multinomial regression model was employed for regressing log-fold change abundances against BARIC values. **d** Log-fold change abundances of Gram-negative microbes relative to Gram-positive microbes across host phenotypes

To assess the predictive capacity of the oral microbiome in stratifying individuals with depressive symptoms, obesity and depressive symptomatology-obesity co-occurrence status, we utilized supervised random forest classification. The prediction performance of the model indicated by both area under the receiver operating characteristic curve (AUROC) and area under precision recall curve (AUPRC), revealed high prediction accuracy (AUROC = 0.75 and AUPRC = 0.74) for obesity-depressive symptom status (Ob/higher Dep) than other groups when multiple samples per-participant were taken into account (Fig. 2a and b). The Ctrl group was predicted with AUROC = 0.75 and AUPRC = 0.58; Ob/lower Dep status with AUROC = 0.70 and AUPRC = 0.49; Non-ob/higher Dep with AUROC = 0.70 and AUPRC = 0.46. However, at sample-level both AUROC and AUPRC ranged from 0.93 to 0.97, across all groups (Fig. S2a and b). Altogether, oral microbiome was highly predictive of depressive symptomatology-obesity co-occurrences than obesity and depressive symptomatology independently.

### Key oral bacterial taxa are associated with specific host phenotype

Next, we identified the most differentially ranked microbes (99 unique taxa) associated with host phenotypes (Fig. 2c). Linear mixed-effects model revealed significant differences in the relative abundances of microbes associated with Ob/higher-Dep (t = 6.5, *p* = 5.07e-08), Non-ob/higher-Dep (t = − 4.2, *p* = 0.0002) and Ob/lower Dep (t = − 4.5, *p* = 5.07e-05) in comparison to Ctrl group, and with inflammation status (t = − 4.83, *p* = 3.03e-05). Most differentially represented taxa (84 unique taxa) were assigned to Gram-negative bacteria such as *Prevotella*, *Aggregatibacter*, *Pseudomonas*, *Campylobacter,* Clostridia *(Selenomonas, Butyrivibrio*, *Veillonella, Megasphaera* and *Schwartzia*), *Leptotrichia, Capnocytophaga*, and periodontal pathogens such as *Treponema*, *Veillonella*, *Porphyromonas* and *Fusobacterium*. Gram-positive (15 unique taxa) were assigned to Peptostreptococcaceae, Clostridia (*Catonella*, Mogibacteriaceae), *Staphylococcus*, *Corynebacterium*, *Rothia*, *Actinomyces*, and beneficial/probiotic genera *Bifidobacterium* and *Lactobacillus* (Fig. 2c, log-fold change abundances for each microbe are shown in Table S1). The Ob/higher-Dep group exhibited a slightly higher abundance of Gram-negative bacteria relative to Gram-positive compared to the Ctrl group (Wilcoxon test: *p* = 0.004) (Fig. 2d), which were not significantly associated with BARIC scores (data not shown).

### Small molecules detected in saliva are associated with obesity-depressive symptom-inflammation relationships

Untargeted liquid chromatography-tandem mass spectrometry (LC-MS/MS) analysis of the saliva samples was performed to examine the metabolic potential in the oral ecosystem and understand the intimate link between salivary microbiota and metabolome in obesity-depressive symptom relationships.

**Fig. 3:**
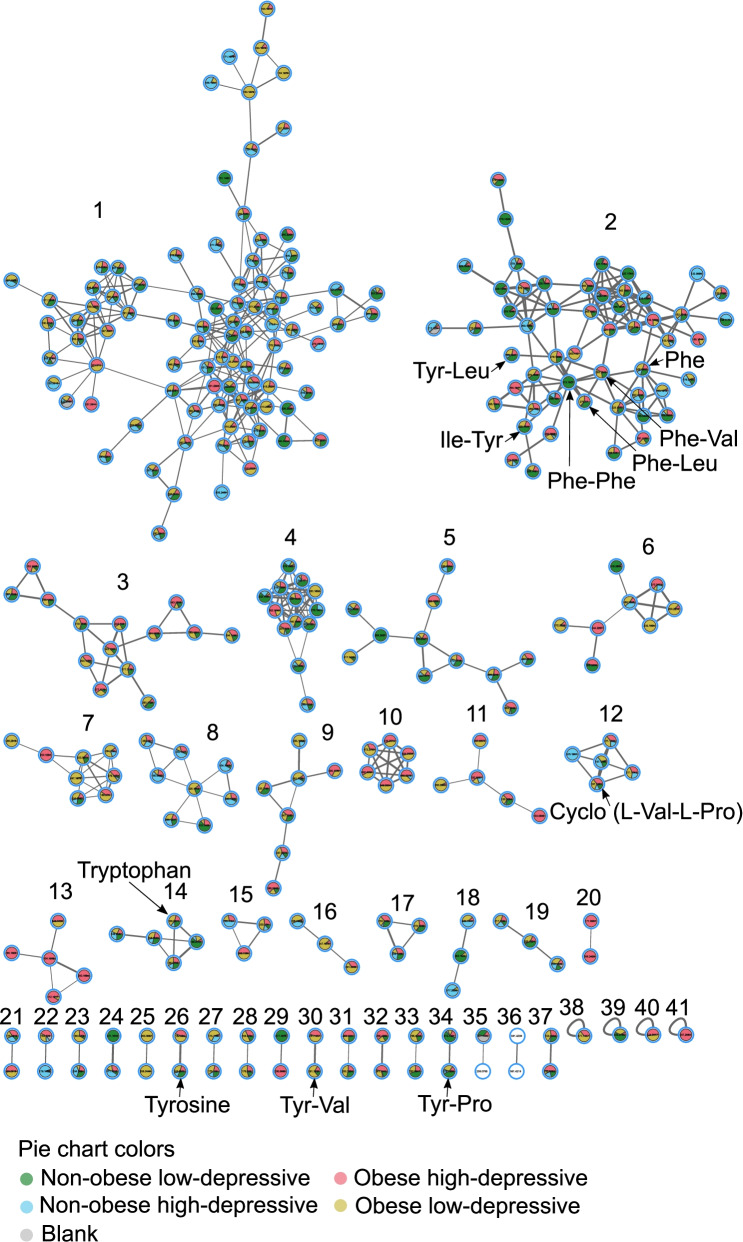
Feature-based molecular network of the ions detected in salivary metabolomes of obese-depressive group. The molecular network was generated by 293 nodes with 41 molecular clusters, which are sub-networks of a larger network generated via Global Natural Products Social Molecular Networking (GNPS). Nodes (small circles with m/z values) represent unique tandem mass spectrometry (MS/MS) consensus spectra and edges (lines) drawn between the nodes correspond to similarity (cosine score) between MS/MS fragmentation. Annotation is performed by MS/MS spectral library matching in GNPS platform. Pie charts within the individual nodes qualitatively represent specific ion presence across groups: non-obese and non-depressive, obese, depressive, and both obese and depressive symptom groups, as well as blank samples. Molecular clusters 2, 3, 4, 5, 9, 17, 19, 30 and 34 represent structural diversity of dipeptides. Molecular clusters 2, 14 and 26 represent aromatic amino acids tryptophan, tyrosine and phenylalanine

**Fig. 4:**
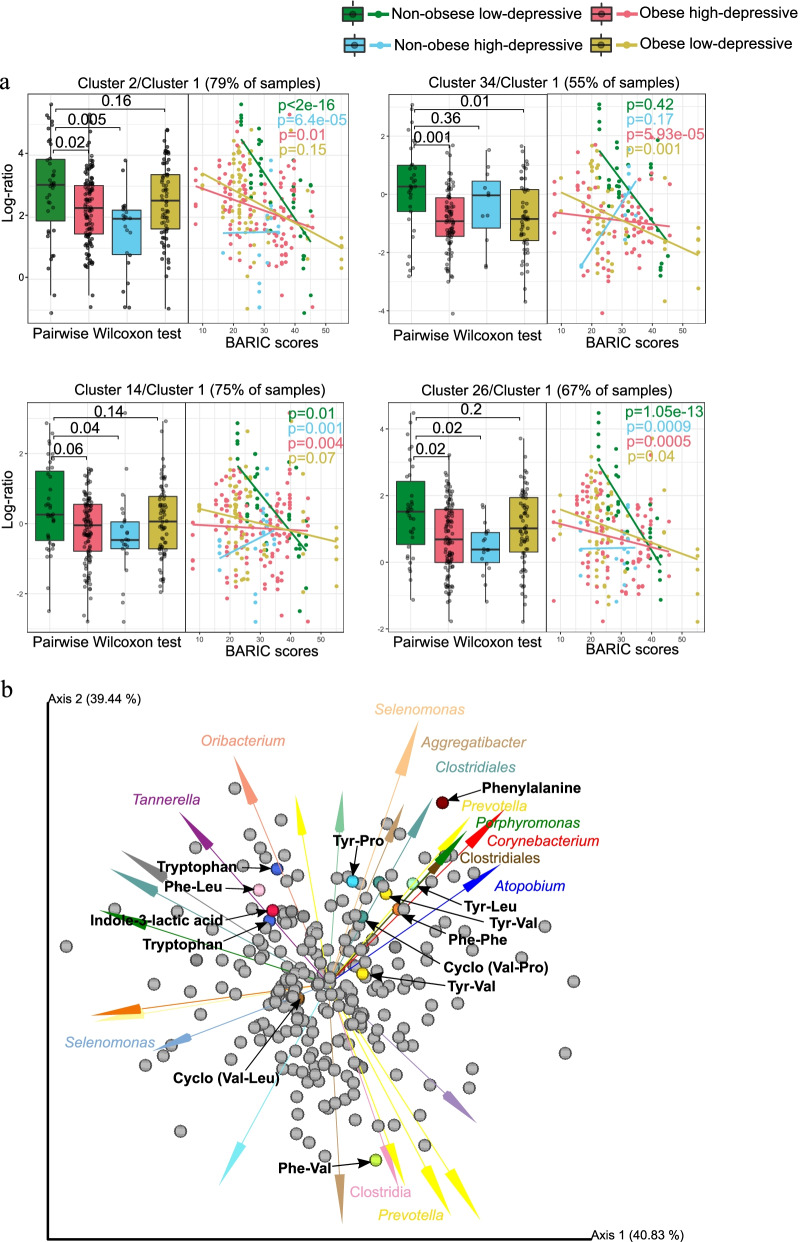
Differentially abundant molecular clusters and microbe-metabolite co-occurrences in obesity-inflammation-depressive and inflammation status. **a** Sample plot showing log-ratio of differential molecular features relative to cluster 1 (see left panel). The corresponding right panels represent a scatterplot of samples showing log-ratio of differential features versus inflammation status. Individual samples are colored by health status. Statistical significance of the log-ratios was evaluated by pairwise comparisons using Wilcoxon rank sum test. A linear regression model was employed for regressing log-ratios against BARIC values. **b** Visualization of microbe-metabolite co-occurrences. Arrows represent microbes and dots represent metabolites. The *x* and *y* axes represent principal components of the microbe-metabolite conditional probabilities as determined by the neural network. Distances between arrow tips quantify co-occurrence strengths between microbes, while directionality of the arrows indicates which microbes and metabolites have a high probability of co-occurring. Only known microbiota-derived molecules are labeled. Microbial abundances are estimated using differential abundance analysis via multinomial regression

The most predominant chemical classes identified from automated chemical classification [46]⁠ of our samples via GNPS [47]⁠ platform were terpenoids, indoles, carbohydrates and carbohydrate conjugates, amino acids, peptides, derivatives of purines and pyrimidines, eicosanoids and linoleic acids (Fig. S3). Particularly, molecular structures of diazines, benzotraizoles, imidazopyrimidines and azides were batch-specific (Fig. S3). Feature-based mass spectral molecular networking of 7818 total MS1 molecular features (which included retention time and relative quantitative information) enabled the annotation of 248 that had matches against all publicly available reference spectra [48]. It should be noted that these are level 2 or 3 annotations according to the 2007 metabolomics standards initiative [49]⁠. A reference-frame based approach enabled the identification of 155 features distinctly associated with specific categories relative to Ctrl group (i.e., Non-Ob/lower-dep) (Fig. 3). Key molecules involved in host-microbiota interactions such as the annotation as tyrosine (level 2), a precursor of catecholamine, dopamine and serotonin, and tryptophan (level 2, cluster 14 and 26 in Fig. 3), a precursor of the neurotransmitter serotonin, were depleted in Ob/higher-Dep and Ob/lower-Dep groups (Fig. 2b). The amino acid, phenylalanine (Level 2, cluster 2 Fig. 3), a biosynthetic precursor of tyrosine, catecholamine, dopa and dopamine was less abundant in the Ob/higher-Dep and Non-ob/higher-Dep groups, but increased with inflammation status (Fig. 4a).

Within the molecular network, we also identified 41 molecular clusters primarily associated with quorum sensing molecules of microbiota, products of microbial transformation of dietary components or host molecules, and essential aromatic amino acids (Fig. 3). Most intriguingly, we identified 34 structurally distinct dipeptides across groups, making it the most prevalent molecular cluster within the network (molecular features of clusters 2, 3, 5, 9, 12, 17, 19, 30, 31, 32 and 34 in Fig. 3). Of these, molecular features of cluster 2 (present in 60 participants) were differentially represented in Ob/higher-Dep and Non-ob/higher-Dep individuals, while features of cluster 34 (present in 58 participants) were differentially represented in Ob/higher-Dep and Ob/lower Dep individuals, when compared to controls (see left panels in Fig. 4a). Moreover, clusters 2, 14 and 26 were depleted in the Ob/higher-Dep and non-ob/higher-Dep groups, while cluster 34 was depleted in the Ob/higher-Dep and Ob/lower Dep groups. Other differentially represented molecular clusters included clusters 14 (detected in 56 participants) and 26 (detected in 58 participants), which encompassed two of the essential aromatic amino acids i.e. tryptophan and tyrosine molecules (see clusters 14 and 26 in Figs. 3 and 4a). Molecular features from these clusters are positively associated with inflammation (right panels in Fig. 4a). Abundance of features from the remaining clusters did not significantly vary across groups (data not shown). Other molecular features included previously reported microbiota-derived dipeptides (Phe-Val and Tyr-Val) (see clusters 2 and 30 in Fig. 3) [42, 50, 51]⁠. Dipeptide (Phe-Phe) reported to be synthesized by *Clostridium* (cluster 2 Fig. 3) [52]⁠ was predominant in the Ob/higher-Dep group. Other molecules such cyclic dipeptides (Val-Pro and Val-Leu), commonly found to be made by microbes, were also identified (see cluster 2 and 12 Figs. 3 and 4a, Table S2) [50, 51]⁠. The majority of the other dipeptides identified were potentially related to host dietary metabolism (i.e. enzymatic digest of food proteins) [43, 44]⁠. Among these, Tyr-Leu, Phe-Leu and Ile-Tyr (cluster 2 Fig. 3), were significantly more abundant in the Ctrl group compared to the other Ob/higher-Dep and Ob/lower-Dep groups (Fig. 4a) among which, Tyr-Pro (cluster 34 Fig. 3) was also depleted (Fig. 4a).

### Key oral microbes co-occurred with biosynthetic precursors of the neurotransmitters and dipeptide signaling molecules

Integration of the microbiome and metabolomics data revealed associations between oral microbial metabolism and key oral microbes such as *Prevotella*, Clostridia, *Selenomonas*, *Aggregatibacter*, *Oribacterium*, *Corynebacterium,* and periodontal pathogens such as *Tannerella* and *Porphyromonas* (Fig. 4b). Dipeptide signaling molecules (Phe-Phe, Phe-Val and Tyr-Val) co-occurred with Clostridia, *Prevotella* and *Porphyromonas*, corroborating known associations of dipeptides produced by *Clostridium* spp. [42, 50–52]⁠. Members of Clostridia also co-occurred with phenylalanine, a potential biosynthetic precursor of dopamine, epinephrine and tryptophan. Intriguingly, *Oribacterium* belonging to *Clostridium* and *Tannerella* co-occurred with tryptophan, shown to encompass tryptophan biosynthetic pathways. Our findings further corroborate known microbial-derived cyclic dipeptides (Val-Leu and Val-Pro) associations with *Selenomonas*, *Aggregatibacter* and *Clostridium* spp. (Fig. 4b) [50, 51]⁠. Potential dietary dipeptides (Phe-Leu, Tyr-Pro and Tyr-Leu) co-occurred with *Tannerella*, *Selenomonas*, *Prevotella*, *Porphyromonas* and Clostridia [43, 44]⁠.

## Discussion

We previously reported that obesity is significantly associated with both inflammation and depressive symptoms [20, 21, 53]⁠. Growing evidence also suggests that gut bacterial composition and their specialized metabolites may trigger chronic systemic inflammation in obesity-depression co-occurrences [2]⁠, highlighting the importance of the host immune and microbial interplay. In this study, we showed that the composition of salivary microbiota differ in co-occurring obesity-depressive symptoms and in relation to obesity, depression, and inflammation. We also showed that individual bacterial taxa were linked to specific host obesity-depressive symptoms ‘phenotype’, and small-molecule mediated microbe-microbe and microbe-host interactions likely play a critical role in these host phenotypes. While effects of obesity, inflammation and depression phenotypes on gut microbiome have been studied previously, this study extends our previous work [33]⁠ that identified relationships between oral microbial composition, host stress profile and inflammatory status, by providing further evidence that oral microbial composition and metabolic profiles are also influenced by the specific host phenotypes, and are likely characterized by significant alterations in the biosynthetic precursors of neurotransmitters and signaling dipeptides. These findings highlight a potential link between oral microbiota and the brain (i.e. oral-brain axis), adding to known gut microbiota-brain interactions [34–36], as well as biomarker utility of oral microbiome in studying brain and behavioral outcomes.

Examining the composition of the oral microbiome revealed significant differences based on obesity, depressive symptomatology and comorbid obesity-depressive symptomatology. At the same time, the oral microbiome composition differed by the host inflammatory processes beyond the effects of obesity or depression. This emphasizes the need of further scrutinizing the central role of microbiome-mediated inflammation in obesity-depressive symptomatology interrelationship and is closely aligned with the existing literature in chronic low-grade inflammation at the intersection of depression and obesity.

Random forest classification indicated that oral microbiota is highly predictive of obesity-depressive symptom co-occurrences, suggesting specific microbial signatures associated with obesity-depression co-occurrences. Corroborating these findings, abundances of several microbes were differentially represented across the obesity-depressive symptomatology groups as revealed by the differential abundance analysis. Gram-negative microbes have been shown to be associated with inflammation due to their LPS cell wall, the hallmark trait of Gram-negative bacteria. We found that Gram-negative microbes *Prevotella*, *Aggregatibacter*, *Pseudomonas*, *Campylobacter*, *Selenomonas*, *Leptotrichia*, *Capnocytophaga*, and Gram-negative periodontal pathogens such as *Treponema*, *Veillonella*, *Porphyromonas* and *Fusobacterium* are enriched in Ob/higher-dep group. However, we found no significant correlation with BARIC scores that measured monocytes’ responsiveness to a β-AR agonist during an inflammatory response to LPS, indicating inflammation regulatory processes [53]. Increased abundance of *Prevotella* in the human oral cavity has been previously ambiguously associated with both health and disease conditions [26, 54, 55]⁠. Pathogenic *Campylobacter* has been shown to increase anxiety-like behavior in mice [56]⁠ and *Aggregatibacter* has been reported to be associated with inflammation. Notably, Gram-positive beneficial microbes *Bifidobacterium* and *Lactobacillus* depleted in Ob/higher-Dep group are in line with their activity as they are reported to exhibit antidepressant and anti-obesity effects, and reduced levels of TNF-α in both clinical and animal studies [57–59]. However, in the present study we found no significant correlation between *Bifidobacterium* and *Lactobacillus* with TNF-α production, suggesting other inflammation regulatory processes [53]⁠. Our findings of Gram-negative and the Gram-positive taxa associated with obesity-depression are in line with recent oral microbiome findings from a study of obesity in a larger cohort [60]⁠. In particular, *Bifidobacterium, Lactobacillus*, *Selemonas*, *Clostridium*, *Porphyromonas and Pseudomonas* association with obesity makes them the most reliable markers of obesity. Future studies should examine the extent to which the oral microbial taxa associated with obesity-depression-inflammation in our study are reciprocated in the gut microbiome within the same individuals or correlate with different gut microbial phenotypes.

We also found differences in relative abundance patterns in many molecules across the obesity-depression symptoms groups, including quorum sensing molecules of microbiota, products of microbial transformation of dietary components or host molecules and aromatic amino acids. Importantly, metabolites of aromatic amino acids tryptophan and tyrosine, both of which are precursors of the neurotransmitter serotonin, have been mechanistically implicated in obesity-depression associations [61]⁠, and play signaling roles in host-microbe interactions in the gut [62]⁠, were depleted in obese individuals compared to the control group. Host dietary dipeptides (Tyr-Leu and Phe-Leu) that were significantly less abundant among the obese individuals compared to the control group in this study are shown to display anti-depressant-like activity as greater abundance of Tyr-Leu activates serotonin, dopamine and gamma aminobutyric acid (GABA) receptors in mice [43, 44]⁠. Tyr-Pro and Ile-Tyr, which were also depleted in the obese individuals in our study, are an inhibitor of angiotensin I-converting enzyme (ACE) with antihypertensive activity [63]⁠ and affect catecholamine (e.g. dopamine and noradrenaline) metabolism in the mouse brain [64]⁠, respectively. These findings offer initial mechanistic insight into comorbid obesity and depression, albeit complex.

Furthermore, we identified several structurally distinct dipeptides that were positively associated with inflammation. To our knowledge, it is the first time that microbial-derived dipeptide (Phe-Val, Tyr-Val and Phe-Phe) and cyclic dipeptides signaling molecules (Val-Pro and Val-Leu) were detected in salivary metabolomes. Biosynthetic gene clusters and the production of dipeptides (Phe-Val and Tyr-Val) have been recently identified in the human microbiome [42, 50, 51]⁠. These molecules are known to play key roles in quorum sensing (cell-to-cell communication to maintain cell density) and virulence, and promote growth of beneficial *Bifidobacterium* [41]⁠. A previous study showed that Phe-Phe derived from *Clostridium* sp. can inhibit host proteins by chemical modification of the host cellular proteins, especially by targeting cathepsins in human cell proteomes [52]. Given our findings that Phe-Phe was highly abundant in the Ob/higher-Dep group, its biological role in the cellular inflammatory process which likely underlie obesity-depression comorbidity warrants further investigation.

Our findings of specific microbe-metabolite interactions with potential to influence host’s brain functioning offer potentially significant insight into the role of host immune-microbiome interplay in comorbid obesity-depression and is likely through microbial neurotransmitters. Metabolic pathways for biosynthesis of neuroactive molecules in the genomes of human-associated genera *Clostridium* and *Tannerella* have been recently reported [35]. Intriguingly, members of *Clostridium* and *Tannerella* co-occurred with tryptophan and have been detected/reported to harbor genes for tryptophan biosynthesis [35]⁠. Members of Clostridia co-occurred with phenylalanine, a potential biosynthetic precursor of dopamine, epinephrine and tryptophan, have been shown to be key species in neuropsychiatric disorders and shown to produce dopamine in mice [36, 65]⁠. While almost all taxa that displayed significant positive correlations with specific neuroactive molecules in the co-occurrence analysis were Gram-negative taxa, beneficial Gram-positive taxa including the genera *Bifidobacterium* and *Lactobacillus*, which were greatly depleted in obese-depressive individuals showed no significant positive co-occurrences with neurotransmitters and neuroactive dipeptides. This suggests that microbial neuroactive molecules modulated through Gram-negative taxa likely play pivotal roles in obesity-inflammation-depression interrelationships. Many of these molecules including the dipeptides, shown to have potential to cross the intestinal barrier and blood brain barrier, may modulate the oral–brain connection through neurotransmitter signaling pathways [35, 65]⁠. Such neurotransmitters and their biosynthetic precursors may offer promising targets for therapeutics.

There are caveats in this study that merit caution: in an effort to recruit individuals with subclinical levels of depressive mood co-occurring with a range of obesity without antidepressant intake or heterogeneous clinical depression, the participants exhibited low levels of BDI scores on average which may limit the applicability of our findings to clinical depression. At the same time, it is notable that host-microbiome-metabolome signatures and their interactions appear to be salient in pathophysiology of subclinical depression symptomatology. We also acknowledge a small sample size of the study participants, despite the expanded specimen sample size owing to multiple saliva collections. Notably, all waking saliva samples were collected prior to oral hygiene activity and ingestion of food or drink, or after an oral rinse with water for non-waking samples. However, additional factors such as diet, oral hygiene practices and oral health status (i.e., dental caries and periodontitis) have been unequivocally shown to impact the oral microbiome, were not recorded in this study. High carbohydrate and sugar consumption associated with a Western diet have been shown to lead to poor oral health as well as obesity, and further studies must be take these into account to gain further understanding of the factors impacting the human oral microbiome in obese-depression interrelationships [66, 67]⁠.

## Conclusions

Despite these limitations, our study significantly expands the evidence for microbial specialized metabolites and peptides with neuroactive potential, adding further research avenues into microbiome-host physiology interactions and there is a great deal of clinical potential in understanding and modifying these interactions. Furthermore, it provides initial evidence for a foundation of the microbial oral-brain axis in addition to the gut-brain axis in the context of obesity-depression-inflammation interrelationships.

## Methods

### Participants

A total of 60 lean to obese participants (20-65 years old) with a range of subclinical depressive symptoms, participating in a larger study investigating the impact of obesity on vascular inflammation and immune cell activation in normotension versus stage 1 hypertension (Basal systolic blood pressure (BP): 130-140 mmHg and diastolic BP: 80-90 mmHg), were included in this study and provided saliva samples. Participant inclusion/exclusion criteria were previously described in detail [33]. Briefly, participants were excluded if they had diabetes, recent history of smoking or substance abuse, history of cardiovascular disease, history of bronchospastic pulmonary disease, inflammatory disorders or health-related factors affecting immune function, psychosis, major depressive disorder, and stage 2 clinical hypertension or with average BP ≥145/90 mmHg measured at the lab visit from six measurements on two separate days, using a Dinamap Compact BP monitor (Critikon, Tampa, FL). Sociodemographic characteristics (i.e., age, sex, and race) and anthropometrics (i.e., height, weight, hip and waist circumference) data were collected.

### Obesity characterization

BMI was calculated based on height and weight measurements (kg/m^2^), and individuals were dichotomized into two groups, based on our prior findings of little notable differences in inflammatory or depressive symptoms state between lean and overweight individuals (ref): non-obese (BMI < 30 kg/m^2^) and obese (BMI ≥30 kg/m^2^). For further adiposity characterization dual x-ray absorptiometry was performed to calculate %total and trunk body fat.

### Depressive symptomatology assessment

Depressive symptoms were assessed using the Beck Depression Inventory (BDI-Ia), a comprehensive and clinically robust self-report 21-item questionnaire (Beck et al., 1996). Each question was scored from 0 to 3, summed to a BDI total score (BDI-T), and then subcategorized into cognitive-affective (BDI-C) and somatic (BDI-S) depression scores based on the items such as BDI-C: guilt, pessimism and BDI-S: fatigue, sleep disruption [68]⁠.

Based on obesity status and mean BDI-T scores, participants were categorized into the following four-groups with a total of 261 saliva samples collected from five time points across the day from 60 participants as described in Supplementary Information (see saliva collection); non-obese and lower-depressive controls (*N* = 10 participants; *n* = 43 samples; “controls”), obese and lower-depressive (*N* = 18; *n* = 74; “Ob/lower-Dep”), non-obese and higher-depressive symptoms (*N* = 5; *n* = 22; “Non-ob/higher-Dep”), and obese and higher-depressive symptoms (*N* = 27; *n* = 122; “Ob/higher-Dep”).

### Blood collection and cellular inflammation assay

For detailed protocol, see Supplementary Materials and Methods section. Briefly, LPS-stimulated blood was incubated with beta-adrenergic receptor agonist isoproterenol and evaluated for intracellular monocyte TNF-α production using flow cytometry, as previously described [53]⁠. Monocyte beta-adrenergic receptor-mediated inflammation control (i.e., “BARIC”, a measure of systemic inflammation) was calculated as the arithmetic difference in %TNF-α-producing monocytes between LPS + media-treated and LPS + isoproterenol-treated samples.

### Saliva collection, DNA extraction and 16S sequencing

For detailed protocols of saliva collection procedure and 16S analysis, see Supplementary Materials and Methods section. Saliva from each participant was collected at five time points across a single day: waking, mid-morning (10:00 h), midday (12:00 h), afternoon (14:00 h), and evening (17:00 h).

### Statistical analyses

Statistical analyses were conducted using R software (version 3.6.3) in RStudio (version 1.2.5019). First, associations among continuous and categorical metadata variables i.e., age, obesity (BMI, %total body fat and trunk fat), BARIC, BDI scores (BDI-T, BDI-C and BDI-S) were assessed using univariate Spearman correlations across all participants using *psych* package in R software. We applied a simple linear mixed-effects model (LMM) fit to model two alpha diversity measures (Shannon index and Faith’s PD) using restricted maximum likelihood (REML) with a random intercept by participant to account for repeated measurements across the day, and main effects of obesity status, depressive symptom status, and BARIC. Age, sex, race were included as covariates in the model. Beta-diversity between groups was tested on the unweighted UniFrac distance metric using non-parametric *PERMANOVA* with distribution-free inferences achieved by 999 permutations for each covariate separately and constrained by participant to adjust for 3-5 samples per participant. A test of homogeneity of dispersion was conducted with the same constraints using *PERMDISP2* procedure with *betadisper* function in *vegan* package to test overall species composition differences within the groups. Next, post-hoc pairwise comparison was performed using *pairwiseAdonis* with Benjamini-Hochberg false discovery rate (FDR) corrections for multiple testing within the *vegan* package in *R.* Alpha level was set to 0.05 for both uncorrected and FDR corrected tests.

### Random forest classifications

A random forest sample classifier was trained based on the 16S data with tuned hyperparameters (num.trees = 500, mtry = 45) in the 20-time repeated, stratified 5-fold cross-validation using *caret* package in R software. The dataset was repeatedly split into five groups with similar class distributions, and we trained the classifier on 80% of the data, and made predictions on the remaining 20% of the data in each fold iteration. We next evaluated the performance of the classifier on predicting the four groups (i.e. controls, Ob/lower-Dep, Non-ob/higher-Dep, Ob/higher-Dep) using both area under the receiver operating characteristic curve (AUROC) and area under the precision-recall curve (AUPRC) based on the samples’ predictions in the holdout test set using *PRROC* package in R. To account for multiple samples per-participant, we next performed 20-time repeated group 3-fold cross-validation, where each participant is in a different testing fold and also samples from the same subjects are never in both testing and training folds.

### Small molecule metabolite detection through mass spectrometry

Saliva was dried and resuspended in 80% MeOH− 20% water and submitted to untargeted LC/MS/MS analysis. For a detailed protocol, see Supplementary Materials and Methods section. To examine the metabolic potential in the oral ecosystem and understand the intimate link between salivary microbiota and metabolome in obesity-depressive symptom relationships, we conducted LC-MS/MS analysis of the saliva samples from the same participants who were first investigated for taxonomic profiling in the above analyses [47, 69]. By integrating feature based molecular networking [70]⁠ with an automated chemical classification [46]⁠ and reference frame based differential abundance analysis [71]⁠ approaches, we revealed differential representation of the key molecular features in obesity and depressive symptom conditions.

### Feature based mass spectral molecular networking (FBMN) and chemically-informed comparison of metabolomic profiles

A data matrix of MS1 features that triggered MS2 scans were uploaded along with the metadata file to Global Natural Product Social Molecular Networking (GNPS) (https://gnps.ucsd.edu) [47]. Feature-based molecular networking (version release_20) [70]⁠ was performed, and library IDs were generated (see Supplementary Materials and Methods section). To further gain a broad overview of the chemistry of salivary metabolomes from MS/MS data, utilizing an automated chemical classification approach [46]⁠, available via GNPS platform, we performed a chemically-informed comparison of untargeted metabolomic profiles across the four groups.

### Differential ranking of taxa and metabolomic features

Differential ranks of taxa and metabolomic features were calculated using Songbird [71]⁠, which uses reference frames. Age, sex, race and time of day of saliva collection were provided as covariates in generating a multinomial regression model based on microbial features. Differential microbial features were visualized alongside de novo phylogenetic tree constructed from the representative sequences of amplicon sequence variants (ASVs) obtained in this study using EMPress [23]⁠. Statistical significance was tested by applying LMMs on log-ratios of the top-and bottom-20 ranked microbes for each group obtained using Qurro rank plots [72]⁠. We applied a linear regression model by utilizing log-ratios of bacterial features and BARIC inflammatory scores to test interactions between obesity-depressive symptoms and inflammation relationships.

To mitigate the inter-batch effect often observed in the metabolomics data due to technical limitations in the number of samples processed in a batch, relative abundances were adjusted for batch specific-effect along with age, sex, race and time of day, utilizing the multivariate model in the reference frame-based approach [71]. We chose cluster 1 (90 features) as the denominator (“reference frame”) for the log-ratio calculations due to its high prevalence across samples, and moreover, GNPS analyses groups structurally similar molecules into a cluster. Statistical significance was tested by applying Friedman test to account for repeated measurements, prior to multiple pairwise comparison analysis using Wilcoxon rank-sum tests with Benjamini-Hochberg FDR corrections for multiple testing.

### Microbe-metabolite interactions through their co-occurrence probabilities

Permutation based differential abundance testing was performed using discrete false-discovery rate correction method [73]⁠ in Calour (https://github.com/biocore/calour) to remove batch-specific MS1 molecular features. Annotated features that were not identified as batch-specific were included in the co-occurrence analysis. Using ASV (*N* = 1516) and annotated molecular features (*N* = 155) as inputs to train neural networks [74]⁠ in QIIME 2 [75]⁠, we estimated the conditional probability that each molecule is present given the presence of a specific microorganism. The resulting conditional probability matrix representing microbe-metabolite interactions was visualized as an EMPeror biplot [74]⁠.

## Supplementary Information

**Additional file 1: **Supplementary Materials and Methods. **Figure S1.** Matrix of plots illustrating Pearson correlations among obesity, depressive symptoms, inflammation and sex, across participants. Histograms of the variables displayed along the matrix diagonal represent distribution of samples and scatter plots of variable pairs are displayed in the off diagonal. Correlation coefficients displayed represent the slopes of the least-squares reference lines in the scatter plots. **Figure S2.** Per sample based RF analysis. (a), Receiver operating characteristic curves (AUROC) illustrating classification accuracy of the random forest model across all groups (i.e. controls, Ob/lower Dep, Non-ob/higher-Dep, Ob/higher-Dep) and (b), Area under precision recall curves (AUPRC) illustrating performance of the random forest model across all groups. **Figure S3.** Chemical diversity captured in salivary metabolomes. Branches in the circular chemical tree are colored according to the class type and branch labels represent putatively annotated chemical features at subclass level based on chemical taxonomy. Bar graphs at the leaf tips illustrate relative abundance of molecules across groups.

**Additional file 2: Supplementary Table 1.** Log-fold change abundances of most differentially ranked microbes across host phenotypes in comparison to the control group. **Supplementary Table 2.** List of metabolites detected in saliva samples of obese, depressed and comorbid obese-depressed adults.

## Data Availability

Sample metadata, the raw and processed 16S sequencing data and their associated feature tables, and preparation metadata are available in Qiita Study ID 11259 (https://qiita.ucsd.edu/study/description/11259). Mass spectral files and LC-MS/MS preparation metadata are accessible from the MassIVE repository accession ID MSV000083077 (ftp://massive.ucsd.edu/MSV000083077). The GNPS feature based molecular networking job is available at https://gnps.ucsd.edu/ProteoSAFe/status.jsp?task=f192a0030f694224a0ba8f08223a1323.

## Abbreviations

IL: Interleukin
TNF-α: Tumor necrosis factor alpha
CRP: C-reactive protein
CNS: Central nervous system
BBB: Blood-brain barrier
LPS: Lipopolysaccharide
AD: Alzheimer’s disease
GABA: Gamma-aminobutyric acid
BDI-T: Beck Depression Inventory total score
BARIC: Monocyte beta-adrenergic receptor-mediated inflammation control
BMI: Body mass index
PCoA: Principal coordinates analysis
AUROC: Area under the receiver operating characteristic curve
AUPRC: Area under precision recall curve
Controls: Non-obese and lower-depressive controls
Ob/lower-Dep: Obese and lower-depressive
Non-ob/higher-Dep: Non-obese and higher-depressive symptoms
Ob/higher-Dep: Obese and higher-depressive symptoms
LC-MS/MS: Liquid chromatography-tandem mass spectrometry
REML: Restricted maximum likelihood
FDR: False discovery rate
FBMN: Feature based mass spectral molecular networking
GNPS: Global Natural Product Social Molecular Networking
ASVs: Amplicon sequence variants

## References

[1] Smith DJ, Court H, McLean G, Martin D, Martin JL, Guthrie B (2014). Depression and multimorbidity: a cross-sectional study of 1,751,841 patients in primary care. J Clin Psychiatry.

[2] Schachter J, Martel J, Lin CS, Chang CJ, Wu TR, Lu CC (2018). Effects of obesity on depression: a role for inflammation and the gut microbiota. Brain Behav Immun.

[3] James SL, Abate D, Abate KH, Abay SM, Abbafati C, Abbasi N (2018). Global, regional, and national incidence, prevalence, and years lived with disability for 354 diseases and injuries for 195 countries and territories, 1990-2017: a systematic analysis for the global burden of disease study 2017. Lancet.

[4] Mannan M, Mamun A, Doi S, Clavarino A (2016). Is there a bi-directional relationship between depression and obesity among adult men and women? Systematic review and bias-adjusted meta analysis. Asian J Psychiatry.

[5] Dawes AJ, Maggard-Gibbons M, Maher AR, Booth MJ, Miake-Lye I, Beroes JM (2016). Mental health conditions among patients seeking and undergoing bariatric surgery a meta-analysis. JAMA.

[6] Pratt LA, Brody DJ. Depression and obesity in the U.S. adult household population, 2005–2010. NCHS data brief, no 167. Hyattsville: National Center for Health Statistics; 2014. 25321386

[7] Luppino FS, De Wit LM, Bouvy PF, Stijnen T, Cuijpers P, Penninx BWJH (2010). Overweight, obesity, and depression: a systematic review and meta-analysis of longitudinal studies. Arch Gen Psychiatry.

[8] Woo YS, Seo HJ, McIntyre RS, Bahk WM (2016). Obesity and its potential effects on antidepressant treatment outcomes in patients with depressive disorders: a literature review. Int J Mol Sci.

[9] Scully T (2014). Public health: society at large. Nature.

[10] Capuron L, Lasselin J, Castanon N (2017). Role of adiposity-driven inflammation in depressive morbidity. Neuropsychopharmacology.

[11] Milano W, Ambrosio P, Carizzone F, De Biasio V, Di Munzio W, Foia MG (2020). Depression and obesity: analysis of common biomarkers. Diseases.

[12] Young JJ, Bruno D, Pomara N (2014). A review of the relationship between proinflammatory cytokines and major depressive disorder. J Affect Disord.

[13] Ouchi N, Parker JL, Lugus JJ, Walsh K (2011). Adipokines in inflammation and metabolic disease. Nat Rev Immunol.

[14] Dalmas E, Clément K, Guerre-Millo M (2011). Defining macrophage phenotype and function in adipose tissue. Trends Immunol.

[15] Wohleb ES, McKim DB, Sheridan JF, Godbout JP. Monocyte trafficking to the brain with stress and inflammation: a novel axis of immune-to-brain communication that influences mood and behavior. Front Neurosci. 2015;8:447. 10.3389/fnins.2014.00447. 10.3389/fnins.2014.00447 PMC4300916 25653581

[16] Miller AH, Raison CL (2016). The role of inflammation in depression: from evolutionary imperative to modern treatment target. Nat Rev Immunol.

[17] Osimo EF, Pillinger T, Rodriguez IM, Khandaker GM, Pariante CM, Howes OD (2020). Inflammatory markers in depression: a meta-analysis of mean differences and variability in 5,166 patients and 5,083 controls. Brain Behav Immun.

[18] Milaneschi Y, Lamers F, Berk M, Penninx BWJH (2020). Depression heterogeneity and its biological underpinnings: toward immunometabolic depression. Biol Psychiatry.

[19] Hong S (2020). Inflammation at the interface of physical and neuropsychiatric outcomes: investigation of neuroendocrine regulatory pathways to inform therapeutics. Brain Behav Immun.

[20] Kohn JN, Cabrera Y, Dimitrov S, Guay-Ross N, Pruitt C, Shaikh FD (2019). Sex-specific roles of cellular inflammation and cardiometabolism in obesity-associated depressive symptomatology. Int J Obes.

[21] Cheng T, Dimitrov S, Pruitt C, Hong S (2016). Glucocorticoid mediated regulation of inflammation in human monocytes is associated with depressive mood and obesity. Psychoneuroendocrinology.

[22] Sharma S, Fulton S (2013). Diet-induced obesity promotes depressive-like behaviour that is associated with neural adaptations in brain reward circuitry. Int J Obes.

[23] Cantrell K, Fedarko MW, Rahman G, McDonald D, Yang Y, Zaw T, et al. EMPress enables tree-guided, interactive, and exploratory analyses of multi-omic data sets. mSystems. 2021;6(2):e01216-20. 10.1128/mSystems.01216-20. 10.1128/mSystems.01216-20 PMC8546999 33727399

[24] Cani PD, Amar J, Iglesias MA, Poggi M, Knauf C, Bastelica D (2007). Metabolic endotoxemia initiates obesity and insulin resistance. Diabetes.

[25] Chen T, Yu WH, Izard J, Baranova OV, Lakshmanan A, Dewhirst FE. The Human Oral Microbiome Database: a web accessible resource for investigating oral microbe taxonomic and genomic information. Database (Oxford). 2010;2010:baq013. 10.1093/database/baq013. 10.1093/database/baq013 PMC2911848 20624719

[26] Dewhirst FE, Chen T, Izard J, Paster BJ, Tanner ACR, Yu WH (2010). The human oral microbiome. J Bacteriol.

[27] Schmidt TSB, Hayward MR, Coelho LP, Li SS, Costea PI, Voigt AY (2019). Extensive transmission of microbes along the gastrointestinal tract. Elife.

[28] Atarashi K, Suda W, Luo C, Kawaguchi T, Motoo I, Narushima S (2017). Ectopic colonization of oral bacteria in the intestine drives TH1 cell induction and inflammation. Science (80-).

[29] Dickson I (2018). Gut microbiota: oral bacteria: a cause of IBD?. Nat Rev Gastroenterol Hepatol.

[30] Dominy SS, Lynch C, Ermini F, Benedyk M, Marczyk A, Konradi A (2019). Porphyromonas gingivalis in Alzheimer’s disease brains: evidence for disease causation and treatment with small-molecule inhibitors. Sci Adv.

[31] Farrokhi V, Nemati R, Nichols FC, Yao X, Anstadt E, Fujiwara M (2013). Bacterial lipodipeptide, lipid 654, is a microbiome-associated biomarker for multiple sclerosis. Clin Transl Immunol.

[32] Shen L (2020). Gut, oral and nasal microbiota and Parkinson’s disease. Microb Cell Factories.

[33] Kohn JN, Kosciolek T, Marotz C, Aleti G, Guay-Ross RN, Hong SH (2020). Differing salivary microbiome diversity, community and diurnal rhythmicity in association with affective state and peripheral inflammation in adults. Brain Behav Immun.

[34] Yano JM, Yu K, Donaldson GP, Shastri GG, Ann P, Ma L (2015). Indigenous bacteria from the gut microbiota regulate host serotonin biosynthesis. Cell.

[35] Valles-Colomer M, Falony G, Darzi Y, Tigchelaar EF, Wang J, Tito RY (2019). The neuroactive potential of the human gut microbiota in quality of life and depression. Nat Microbiol.

[36] Olsen I, Hicks SD. Oral microbiota and autism spectrum disorder (ASD). J Oral Microbiol. 2019;12(1):1702806. 10.1080/20002297.2019.1702806. 10.1080/20002297.2019.1702806 PMC6913665 31893019

[37] Olsen I (2008). Update on bacteraemia related to dental procedures. Transfus Apher Sci.

[38] Aleti G, Baker JL, Tang X, Alvarez R, Dinis M, Tran NC (2019). Identification of the bacterial biosynthetic gene clusters of the oral microbiome illuminates the unexplored social language of bacteria during health and disease. MBio.

[39] Garg N, Luzzatto-Knaan T, Melnik AV, Caraballo-Rodríguez AM, Floros DJ, Petras D (2017). Natural products as mediators of disease. Nat Prod Rep.

[40] Donia MS, Fischbach MA (2015). Small molecules from the human microbiota. Science (80-).

[41] Hatanaka M, Morita H, Aoyagi Y, Sasaki K, Sasaki D, Kondo A (2020). Effective bifidogenic growth factors cyclo-Val-Leu and cyclo-Val-Ile produced by Bacillus subtilis C-3102 in the human colonic microbiota model. Sci Rep.

[42] Cao L, Shcherbin E, Mohimani H (2019). A metabolome- and metagenome-wide association network reveals microbial natural products and microbial biotransformation products from the human microbiota. mSystems.

[43] Mizushige T, Uchida T, Ohinata K (2020). Dipeptide tyrosyl-leucine exhibits antidepressant-like activity in mice. Sci Rep.

[44] Kanegawa N, Suzuki C, Ohinata K (2010). Dipeptide Tyr-Leu (YL) exhibits anxiolytic-like activity after oral administration via activating serotonin 5-HT1A, dopamine D1 and GABAA receptors in mice. FEBS Lett.

[45] Franzosa EA, Sirota-Madi A, Avila-Pacheco J, Fornelos N, Haiser HJ, Reinker S (2019). Gut microbiome structure and metabolic activity in inflammatory bowel disease. Nat Microbiol.

[46] Tripathi A, Vázquez-Baeza Y, Gauglitz JM, Wang M, Dührkop K, Nothias-Esposito M (2021). Chemically informed analyses of metabolomics mass spectrometry data with Qemistree. Nat Chem Biol.

[47] Wang M, Carver JJ, Phelan VV, Sanchez LM, Garg N, Peng Y (2016). Sharing and community curation of mass spectrometry data with Global Natural Products Social Molecular Networking. Nat Biotechnol.

[48] Aksenov AA, Da Silva R, Knight R, Lopes NP, Dorrestein PC (2017). Global chemical analysis of biology by mass spectrometry. Nat Rev Chem.

[49] Sumner LW, Amberg A, Barrett D, Beale MH, Beger R, Daykin CA (2007). Proposed minimum reporting standards for chemical analysis: Chemical Analysis Working Group (CAWG) Metabolomics Standards Initiative (MSI). Metabolomics.

[50] Park HB, Crawford JM (2016). Pyrazinone protease inhibitor metabolites from Photorhabdus luminescens. J Antibiot (Tokyo).

[51] Wyatt MA, Mok MCY, Junop M, Magarvey NA (2012). Heterologous expression and structural characterisation of a pyrazinone natural product assembly line. ChemBioChem.

[52] Guo CJ, Chang FY, Wyche TP, Backus KM, Acker TM, Funabashi M (2017). Discovery of reactive microbiota-derived metabolites that inhibit host proteases. Cell.

[53] Hong S, Dimitrov S, Cheng T, Redwine L, Pruitt C, Mills PJ (2015). Beta-adrenergic receptor mediated inflammation control by monocytes is associated with blood pressure and risk factors for cardiovascular disease. Brain Behav Immun.

[54] Zhang L, Liu Y, Zheng HJ, Zhang CP (2020). The oral microbiota may have influence on oral cancer. Front Cell Infect Microbiol.

[55] Larsen JM (2017). The immune response to Prevotella bacteria in chronic inflammatory disease. Immunology.

[56] Goehler LE, Park SM, Opitz N, Lyte M, Gaykema RPA (2008). Campylobacter jejuni infection increases anxiety-like behavior in the holeboard: possible anatomical substrates for viscerosensory modulation of exploratory behavior. Brain Behav Immun.

[57] Abildgaard A, Elfving B, Hokland M, Lund S, Wegener G (2017). Probiotic treatment protects against the pro-depressant-like effect of high-fat diet in Flinders Sensitive Line rats. Brain Behav Immun.

[58] Abildgaard A, Elfving B, Hokland M, Wegener G, Lund S (2017). Probiotic treatment reduces depressive-like behaviour in rats independently of diet. Psychoneuroendocrinology.

[59] Schellekens H, Torres-Fuentes C, van de Wouw M, Long-Smith CM, Mitchell A, Strain C (2021). Bifidobacterium longum counters the effects of obesity: partial successful translation from rodent to human. EBioMedicine.

[60] Yang Y, Cai Q, Zheng W, Steinwandel M, Blot WJ, Shu XO (2019). Oral microbiome and obesity in a large study of low-income and African-American populations. J Oral Microbiol.

[61] Chaves Filho AJM, Lima CNC, Vasconcelos SMM, de Lucena DF, Maes M, Macedo D (2018). IDO chronic immune activation and tryptophan metabolic pathway: a potential pathophysiological link between depression and obesity. Prog Neuropsychopharmacol Biol Psychiatry.

[62] Roager HM, Licht TR (2018). Microbial tryptophan catabolites in health and disease. Nat Commun.

[63] Yamamoto N, Maeno M, Takano T (1999). Purification and characterization of an antihypertensive peptide from a yogurt-like product fermented by Lactobacillus helveticus CPN4. J Dairy Sci.

[64] Moriyasu K, Ichinose T, Nakahata A, Tanaka M, Matsui T, Furuya S (2016). The dipeptides Ile-Tyr and Ser-Tyr exert distinct effects on catecholamine metabolism in the mouse brainstem. Int J Pept.

[65] Asano Y, Hiramoto T, Nishino R, Aiba Y, Kimura T, Yoshihara K (2012). Critical role of gut microbiota in the production of biologically active, free catecholamines in the gut lumen of mice. Am J Physiol Gastrointest Liver Physiol.

[66] Adler CJ, Dobney K, Weyrich LS, Kaidonis J, Walker AW, Haak W (2013). Sequencing ancient calcified dental plaque shows changes in oral microbiota with dietary shifts of the Neolithic and industrial revolutions. Nat Genet.

[67] Goodson JM, Hartman ML, Shi P, Hasturk H, Yaskell T, Vargas J (2017). The salivary microbiome is altered in the presence of a high salivary glucose concentration. PLoS One.

[68] Beck AT, Steer RA, Ball R, Ranieri WF (1996). Comparison of Beck depression inventories -IA and -II in psychiatric outpatients. J Pers Assess.

[69] Aron AT, Gentry EC, McPhail KL, Nothias LF, Nothias-Esposito M, Bouslimani A (2020). Reproducible molecular networking of untargeted mass spectrometry data using GNPS. Nat Protoc.

[70] Nothias LF, Petras D, Schmid R, Dührkop K, Rainer J, Sarvepalli A (2020). Feature-based molecular networking in the GNPS analysis environment. Nat Methods.

[71] Morton JT, Marotz C, Washburne A, Silverman J, Zaramela LS, Edlund A (2019). Establishing microbial composition measurement standards with reference frames. Nat Commun.

[72] Fedarko MW, Martino C, Morton JT, González A, Rahman G, Marotz CA (2020). Visualizing ‘omic feature rankings and log-ratios using Qurro. NAR Genomics Bioinform.

[73] Jiang L, Amir A, Morton JT, Heller R, Arias-Castro E, Knight R (2017). Discrete false-discovery rate improves identification of differentially abundant microbes. mSystems.

[74] Morton JT, Aksenov AA, Nothias LF, Foulds JR, Quinn RA, Badri MH (2019). Learning representations of microbe–metabolite interactions. Nat Methods.

[75] Bolyen E, Rideout JR, Dillon MR, Bokulich NA, Abnet CC, Al-Ghalith GA (2019). Reproducible, interactive, scalable and extensible microbiome data science using QIIME 2. Nat Biotechnol.

