# Supplementary material for: Salivary bacterial signatures in depression-obesity comorbidity are associated with neurotransmitters and neuroactive dipeptides
Source: BMC Microbiol. 2022 Mar 14;22:75. doi: 10.1186/s12866-022-02483-4 (PMC8919597; doi:10.1186/s12866-022-02483-4)
Supplement: Supplementary file 1 — Additional file 1: Supplementary Materials and Methods. Figure S1. Matrix of plots illustrating Pearson correlations among obesity, depressive symptoms, inflammation and sex, across participants. Histograms of the variables displayed along the matrix diagonal represent distribution of samples and scatter plots of variable pairs are displayed in the off diagonal. Correlation coefficients displayed represent the slopes of the least-squares reference lines in the scatter plots. Figure S2. Per sample based RF analysis. (a), Receiver operating characteristic curves (AUROC) illustrating classification accuracy of the random forest model across all groups (i.e. controls, Ob/lower Dep, Non-ob/higher-Dep, Ob/higher-Dep) and (b), Area under precision recall curves (AUPRC) illustrating performance of the random forest model across all groups. Figure S3. Chemical diversity captured in salivary metabolomes. Branches in the circular chemical tree are colored according to the class type and branch labels represent putatively annotated chemical features at subclass level based on chemical taxonomy. Bar graphs at the leaf tips illustrate relative abundance of molecules across groups. [file 12866_2022_2483_MOESM1_ESM.zip › Supplementary Figure 3.pdf]

Fig. S3

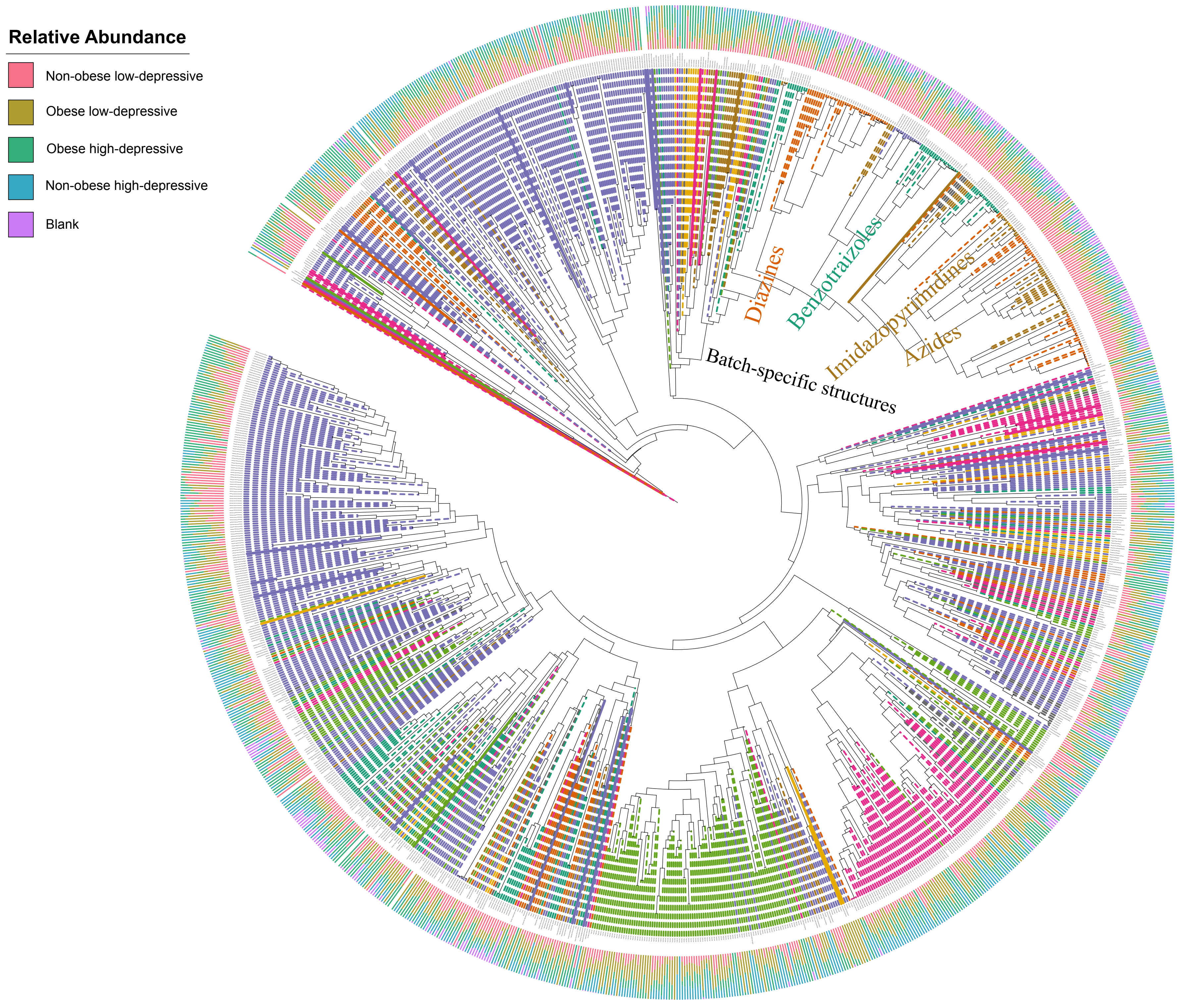
Relative Abundance
Non-obese low-depressive
Obese low-depressive
Obese high-depressive
Non-obese high-depressive
Blank
Diazines
Benzotraizoles
Imidazopyrimidines
Azides
Batch-specific structures
