# Supplementary material for: Salivary bacterial signatures in depression-obesity comorbidity are associated with neurotransmitters and neuroactive dipeptides
Source: BMC Microbiol. 2022 Mar 14;22:75. doi: 10.1186/s12866-022-02483-4 (PMC8919597; doi:10.1186/s12866-022-02483-4)
Supplement: Supplementary file 1 — Additional file 1: Supplementary Materials and Methods. Figure S1. Matrix of plots illustrating Pearson correlations among obesity, depressive symptoms, inflammation and sex, across participants. Histograms of the variables displayed along the matrix diagonal represent distribution of samples and scatter plots of variable pairs are displayed in the off diagonal. Correlation coefficients displayed represent the slopes of the least-squares reference lines in the scatter plots. Figure S2. Per sample based RF analysis. (a), Receiver operating characteristic curves (AUROC) illustrating classification accuracy of the random forest model across all groups (i.e. controls, Ob/lower Dep, Non-ob/higher-Dep, Ob/higher-Dep) and (b), Area under precision recall curves (AUPRC) illustrating performance of the random forest model across all groups. Figure S3. Chemical diversity captured in salivary metabolomes. Branches in the circular chemical tree are colored according to the class type and branch labels represent putatively annotated chemical features at subclass level based on chemical taxonomy. Bar graphs at the leaf tips illustrate relative abundance of molecules across groups. [file 12866_2022_2483_MOESM1_ESM.zip › supplementary_material_01242022 submit.docx]

**Supplementary Materials and Methods**

**Blood collection and cellular inflammation assay**

Blood samples were obtained for all participants after 12h of fasting except for plain water and collected in heparin anti-coagulant vacutainers (BD, Franklin Lakes, NJ). Cellular inflammation regulation assays were performed on heparinized whole blood within 1h of collection. Briefly, 200 pg/mL of lipopolysaccharide (LPS) (E.coli 0111:B4, catalog #L4391, Sigma-Aldrich, St. Louis, MO) was added to 300 μL of blood in sterile 96-well polypropylene cell culture plates and incubated for 30 min at 37°C with 5% CO_2_. Media-treated samples served as controls. This exogenous LPS dose was previously determined to elicit significant activation of monocytes, with 30-90% producing TNF-α (1)⁠. Monocyte beta-adrenergic receptor-mediated inflammation control (i.e., “BARIC”) was determined based on the inhibitory effect of isoproterenol (Iso), a non-specific β1/2AR agonist, on monocytic intracellular TNF-α production in LPS-stimulated blood as aforementioned. Briefly, LPS-stimulated blood was incubated with isoproterenol in 10^-8^ M final concentration and evaluated for intracellular monocyte TNF-α production using flow cytometry, as previously described (1)⁠. The proportion of CD14^+/dim^HLA-DR^+^ (CD14: cat. #301808; HLA-DR: cat. #307606, BioLegend, San Diego, CA) cells that were TNF-α^+^ was determined using FlowJo software (v10, TreeStar, Ashland, OR), and gates adjusted for TNF-α-stained sample via fluorescence-minus-one controls (2,3)⁠. Ultimately, BARIC was calculated as the arithmetic difference in %TNF-α+ monocytes between LPS-treated and LPS+isoproterenol-treated samples. Greater BARIC values indicate greater β-AR responsivity, and thus, better Iso/β-AR-mediated inflammation regulation. Smaller BARIC values may indicate impairment in cellular pathways that regulate inflammatory responses mediated by β-ARs (e.g., diminished receptor sensitivity to agonists). BARIC measures monocytes responsivity to a β-AR agonist during an inflammatory response to LPS. Reduced BARIC has been associated with hypertension, cardiovascular disease risk factors, obesity, and higher serum cytokine levels (2,3)⁠.

**Saliva collection, DNA extraction and 16S sequencing**

Saliva collection procedure and 16S sequencing data was published previously (2)⁠. However, obesity-depressive symptom relationships were not previously investigated, and instead had focused on temporal variation of the oral microbiota. Briefly, participants were provided with Salivette (Sarstedt, #51.1534, Nümbrecht, Germany) to roll the cotton Salivette inside the mouth to stimulate salivation without chewing. Saturated Salivette was placed back into the tube by mouth. Salivettes from each participant were collected at five time points across a single day: waking, mid-morning (10:00 hrs), midday (12:00 hrs), afternoon (14:00 hrs), and evening (17:00 hr). All waking samples were collected prior to oral hygiene activity, and ingestion of food or drink. In addition, participants were instructed to abstain from consuming food or drinks other than plain water for 30 min and to rinse their mouth with water prior to collection at all other time points. Next, saliva was recovered from Salivette tubes by centrifuging at 1,000 x g for 2 minutes at 4°C and stored at -80°C. DNA from saliva samples was extracted by employing Qiagen PowerSoil DNA kit as previously described (4). V4 region of the 16S gene was amplified according to the Earth Microbiome Project protocol (5,6)⁠ and sequenced on the Illumina MiSeq sequencing platform with a MiSeq Reagent Kit v2 and paired-end 150 bp cycles.

**16S sequencing data processing**

Sequences were demultiplexed based on the barcode associated with each sample and sequence quality control and ASV (Amplicon Sequence Variants) feature table construction was conducted using the Deblur algorithm in QIIME2 (v.2018.4) (7). The resulting Greengenes 13_8 database hit table contained sequences that have been filtered to exclude non-16S sequences such as organellar reads from mitochondria and chloroplast. Taxonomy assignment was performed by employing the QIIME2 feature-classifier plugin with a pre-fit classifier (8)⁠ for the 99% reference tree of Greengenes 13_8 database, implemented in Qiita (9)⁠. Next, 223 potential sequencing contaminants that appeared in both true and blank samples were removed from the ASV table using *decontam* in R (10)⁠. Feature table was filtered using QIIME2 to remove low abundance features with fewer than 10 reads across samples and singleton features present only in one sample. This final feature table contained 1,516 unique features/ASVs and 455 unique taxa with an average of 19,412 ± 9,187 sequences per sample. Next, alpha-diversity indices, including the Shannon diversity index and Faith’s Phylogenetic Diversity were calculated by rarefying the ASV table at a minimum sequencing depth of 1,122 reads per sample to mitigate uneven sequencing depth across samples. Four samples with total reads below the defined threshold were excluded during the rarefaction. Beta-diversity, was calculated on the unrarefied feature table using unweighted UniFrac distance, which reflects presence-absence of taxa. We performed ordination on output distance matrices using principal coordinates analysis (PCoA) and following visualization using EMPeror plugin in QIIME2 (11)⁠.

**Small molecule metabolites detection through mass spectrometry**

Saliva was dried and resuspended in 80% MeOH−20% water (Optima LC-MS grade; Fisher Scientific, Fair Lawn, NJ, USA). Untargeted metabolomics was conducted with an ultrahigh-performance liquid chromatography (Vanquish; Thermo Fisher Scientific, Waltham, MA, USA) system coupled to an orbitrap mass spectrometer (QExactive, Thermo Fisher Scientific). A C18 reversed-phase UHPLC column (Kinetex, 1.7-µm particles size, 50 x 2.1 mm) (Phenomenex, Torrance, CA, USA) was used for chromatographic separation. A linear gradient was applied as follows: 0 to 0.5 min, isocratic at 5% mobile phase (MP) B; 0.5 to 8.5 min, 100% MP B; 8.5 to 11 min, isocratic at 100% MP B; 11 to 11.5 min, 5% MP B; 11.5 to 12 min, 5% MP B, where mobile phase A is water with 0.1% formic acid (vol/vol) and mobile phase B is acetonitrile−0.1% formic acid (vol/vol) (LC-MS grade solvents; Fisher Chemical). Electrospray ionization in the positive mode was used. MS spectra were acquired in the mass range of m/z 100 to 2,000.

**MS1 feature finding and data processing**

Raw QExactive files were converted to .mzXML format using ProteoWizard tool MSConvert (12)⁠ software. Data quality was assessed by evaluating the m/z error and retention time of the LC-MS standard solution (i.e., mixture of six compounds). MS1 feature finding was performed in MZmine2 preprocessing workflow (MZmine-2.37.corr17.7_kai_merge2 version) available at (https://github.com/robinschmid/mzmine2/releases) (13)⁠. The mzMINE parameters used for feature finding are as follows: mass detection (centroid; MS1, 1.5E3; MS2, 90); ADAP Chromatogram builder (minimum group size in number of scans, 4; group intensity threshold, 5E3; minimum highest intensity, 2E3; m/z tolerance, 0.001 m/z to 20 ppm); chromatogram deconvolution (local minimum search, chromatographic threshold of 96%, search minimum in retention time [RT] range [minutes] of 0.03, minimum relative height of 5%, minimum absolute height of 2E3, minimum ratio of peak top/edge of 1 and peak duration range [minutes] of 0 to 2;m/z center calculation set to auto; m/z range for MS2 scan pairing (daltons) of 0.02 and RT range for MS2 scan pairing (minutes) of 0.15); isotope peaks grouper (m/z tolerance set to 0.0015 m/z or 10 ppm; retention time tolerance of 0.05, maximum charge of 3; and representative isotope set to most intense); order peak lists; join aligner (m/z tolerance set at 0.0015 m/z or 15 ppm; weight for m/z of 2; retention time tolerance of 0.2 min; weight for RT of 1. A filter was used such that only features present in at least two samples were included.

**Feature based mass spectral molecular networking (FBMN)**

The output of aforementioned workflow, a data matrix of MS1 features that triggered MS2 scans by sample (.mgf and .csv quant table), were uploaded along with the metadata file to Global Natural Product Social Molecular Networking (GNPS) (https://gnps.ucsd.edu) (14,15)⁠. Feature-based molecular networking (version release_20) (16)⁠ was performed, and library IDs were generated. Molecular networking parameters were set as follows: precursor ion mass tolerance and fragment ion tolerance of 0.02 Da to cluster consensus spectra; the minimum score between a pair of MS2 consensus spectra was set at 0.7 and 6 as the minimum number of ions matched as described at https://gnps.ucsd.edu/ProteoSAFe/status.jsp?task=f192a0030f694224a0ba8f08223a1323. The molecular network output from GNPS was then uploaded to Cytoscape (version 3.5.1 http://www.cytoscape.org/) (17)⁠, for advanced visualization. Nodes were labelled with spectral matches to GNPS with m/z values, and edge thickness is proportional to the cosine score.

**Supplementary figures**

**Figure S1**. Matrix of plots illustrating Pearson correlations among obesity, depressive symptoms, inflammation and sex, across participants. Histograms of the variables displayed along the matrix diagonal represent distribution of samples and scatter plots of variable pairs are displayed in the off diagonal. Correlation coefficients displayed represent the slopes of the least-squares reference lines in the scatter plots.

**Figure S2**. Per sample based RF analysis. (a), Receiver operating characteristic curves (AUROC) illustrating classification accuracy of the random forest model across all groups (i.e. controls, Ob/lower Dep, Non-ob/higher-Dep, Ob/higher-Dep) and (b), Area under precision recall curves (AUPRC) illustrating performance of the random forest model across all groups.

**Figure S3**. Chemical diversity captured in salivary metabolomes. Branches in the circular chemical tree are colored according to the class type and branch labels represent putatively annotated chemical features at subclass level based on chemical taxonomy. Bar graphs at the leaf tips illustrate relative abundance of molecules across groups.

**References**

1. Hong S, Dimitrov S, Cheng T, Redwine L, Pruitt C, Mills PJ, et al. Beta-adrenergic receptor mediated inflammation control by monocytes is associated with blood pressure and risk factors for cardiovascular disease. Brain Behav Immun. 2015 Nov 1;50:31–8.

2. Kohn JN, Cabrera Y, Dimitrov S, Guay-Ross N, Pruitt C, Shaikh FD, et al. Sex-specific roles of cellular inflammation and cardiometabolism in obesity-associated depressive symptomatology. Int J Obes. 2019 Oct 1;43(10):2045–56.

3. Dimitrov S, Hulteng E, Hong S. Inflammation and exercise: Inhibition of monocytic intracellular TNF production by acute exercise via β2-adrenergic activation. Brain Behav Immun. 2017 Mar 1;61:60–8.

4. Marotz C, Amir A, Humphrey G, Gaffney J, Gogul G, Knight R. DNA extraction for streamlined metagenomics of diverse environmental samples. Biotechniques. 2017;62(6):290–3.

5. Thompson LR, Sanders JG, McDonald D, Amir A, Ladau J, Locey KJ, et al. A communal catalogue reveals Earth’s multiscale microbial diversity. Nature. 2017 Nov 23;551(7681):457–63.

6. Walters W, Hyde ER, Berg-Lyons D, Ackermann G, Humphrey G, Parada A, et al. Improved Bacterial 16S rRNA Gene (V4 and V4-5) and Fungal Internal Transcribed Spacer Marker Gene Primers for Microbial Community Surveys. mSystems. 2016 Feb 25;1(1):9–15.

7. Bolyen E, Rideout JR, Dillon MR, Bokulich NA, Abnet CC, Al-Ghalith GA, et al. Reproducible, interactive, scalable and extensible microbiome data science using QIIME 2. Vol. 37, Nature Biotechnology. Nature Publishing Group; 2019. p. 852–7.

8. Bokulich NA, Kaehler BD, Rideout JR, Dillon M, Bolyen E, Knight R, et al. Optimizing taxonomic classification of marker-gene amplicon sequences with QIIME 2’s q2-feature-classifier plugin. Microbiome. 2018 May 17;6(1).

9. Gonzalez A, Navas-Molina JA, Kosciolek T, McDonald D, Vázquez-Baeza Y, Ackermann G, et al. Qiita: rapid, web-enabled microbiome meta-analysis. Nat Methods. 2018 Oct 1;15(10):796–8.

10. Davis NM, Proctor DiM, Holmes SP, Relman DA, Callahan BJ. Simple statistical identification and removal of contaminant sequences in marker-gene and metagenomics data. Microbiome. 2018 Dec 17;6(1).

11. Vázquez-Baeza Y, Pirrung M, Gonzalez A, Knight R. EMPeror: A tool for visualizing high-throughput microbial community data. Gigascience. 2013;2(1).

12. Chambers MC, MacLean B, Burke R, Amodei D, Ruderman DL, Neumann S, et al. A cross-platform toolkit for mass spectrometry and proteomics. Vol. 30, Nature Biotechnology. Nat Biotechnol; 2012. p. 918–20.

13. Pluskal T, Castillo S, Villar-Briones A, Orešič M. MZmine 2: Modular framework for processing, visualizing, and analyzing mass spectrometry-based molecular profile data. BMC Bioinformatics. 2010 Jul 23;11.

14. Aron AT, Gentry EC, McPhail KL, Nothias LF, Nothias-Esposito M, Bouslimani A, et al. Reproducible molecular networking of untargeted mass spectrometry data using GNPS. Nat Protoc. 2020 Jun 1;15(6):1954–91.

15. Wang M, Carver JJ, Phelan V V., Sanchez LM, Garg N, Peng Y, et al. Sharing and community curation of mass spectrometry data with Global Natural Products Social Molecular Networking. Vol. 34, Nature Biotechnology. Nature Publishing Group; 2016. p. 828–37.

16. Nothias LF, Petras D, Schmid R, Dührkop K, Rainer J, Sarvepalli A, et al. Feature-based molecular networking in the GNPS analysis environment. Nat Methods. 2020 Sep 1;17(9):905–8.

17. Shannon P, Markiel A, Ozier O, Baliga NS, Wang JT, Ramage D, et al. Cytoscape: A software Environment for integrated models of biomolecular interaction networks. Genome Res. 2003 Nov;13(11):2498–504.
